# Fifteen‐year survival and conditional survival of women with breast cancer in Osaka, Japan: A population‐based study

**DOI:** 10.1002/cam4.6016

**Published:** 2023-05-04

**Authors:** Mizuki Kato, Kayo Nakata, Toshitaka Morishima, Yoshihiro Kuwabara, Fumie Fujisawa, Nobuyoshi Kittaka, Takahiro Nakayama, Isao Miyashiro

**Affiliations:** ^1^ Cancer Control Center, Osaka International Cancer Institute Osaka Japan; ^2^ Department of Medical Oncology Osaka International Cancer Institute Osaka Japan; ^3^ Department of Breast Surgery Osaka Rosai Hospital Osaka Japan; ^4^ Department of Breast and Endocrine Surgery Osaka International Cancer Institute Osaka Japan

**Keywords:** breast cancer, epidemiology, survival, women's cancer

## Abstract

**Background:**

In recent years, the survival of patients with breast cancer has improved. However, few published studies have a longer than 10‐year follow‐up. Conditional relative survival (CRS), which is relative survival (RS) of patients who have survived beyond a certain period after diagnosis, is useful for assessing excess mortality among long‐term survivors compared with the general population.

**Methods:**

This was a retrospective observational cohort study. Population‐based cancer registry data in Osaka, Japan were used to determine 15‐year RS and 5‐year CRS of women with breast cancer diagnosed between 2001 and 2002 and followed up for at least 15 years. Fifteen‐year RS and age‐standardized RS (ASR) were calculated by Ederer II and cohort methods. Five‐year CRS according to age group and extent of disease (localized, regional, and distant) was estimated for every year from diagnosis to 10 years.

**Results:**

In the cohort of 4006 patients, the ASR declined progressively, the 5‐year ASR being 85.8%, 10‐year ASR 77.3%, and 15‐year ASR 71.6%. The overall 5‐year CRS exceeded 90% at 5 years after diagnosis, reflecting a small excess mortality compared with the general population. The 5‐year CRS of patients with regional and distant disease did not reach 90% within 10 years of follow‐up (89.4% for regional and 72.9% for distant disease 10 years after diagnosis), indicating that these patients had substantial excess mortality.

**Conclusion:**

Long‐term survival data can help cancer survivors plan their lives and receive better medical care and support.

## 1 INTRODUCTION

Breast cancer is common in many parts of the world. In 2020, approximately 2.2 million new cases were reported worldwide,^1^ accounting for approximately one‐fourth of all cancers in women and girls. In Japan, there were 93,858 new diagnoses of breast cancer in 2018,^2^ accounting for 22.2% of all female patients with cancer. It was the most common cancer in women and its incidence is increasing.^3^

An improvement in survival of patients with breast cancer has been demonstrated in many areas of the world.^4^, ^5^, ^6^, ^7^, ^8^ The 10‐year relative survival (RS) improved from 76.9% in 1993–1997 to 79.3% in 2002–2006 in Japan.^9^ However, late recurrence, even after completion of 5‐years of treatment, has been reported.^10^ Accordingly, the duration of breast cancer treatment has become longer. In 2019, the American Society of Clinical Oncology Clinical Practice Guideline recommended continuing adjuvant endocrine therapy for 10 years.^11^

Even though many patients with breast cancer now survive longer, few population‐based studies with observations beyond 10 years have been published.^9^, ^12^ Information on actual prognosis is very helpful for life planning by patients with cancer, especially long‐term survivors.^13^ The conditional relative survival (CRS) indicates the prognosis of patients who have survived beyond a certain period of time. When this rate is 100%, there is no excess mortality compared with the general population. Calculating these rates enables assessment of excess mortality of patients with breast cancer. In this study, we drew on a population‐based cancer registry data in Osaka to analyze 15‐year RS and 5‐year CRS of 0‐ to 10‐year survivors of breast cancer. We also performed stratified analyses to determine how survival and excess mortality changed with time from diagnosis and how they varied by patient age and extent of disease.

## 2 METHODS

### 2.1 Study design and data source

This was a retrospective observational cohort study using population‐based cancer registry data from the Osaka Cancer Registry (OCR). The OCR, established in 1962, is one of the longest‐established population‐based cancer registries in Japan, covering all cancers diagnosed in Osaka Prefecture (population 8.8 million [2002 census]; 7.0% of the Japanese population). The following information is included in the OCR: International Classification of Diseases, 10th Edition (ICD‐10) codes, extent of disease, age at diagnosis, year and month of diagnosis, and date of death or the last date of confirmation of vital status for each patient. The OCR routinely checks the vital status by death certificate every year and by checking the inhabitant's registry at 3, 5, and 10 years after diagnosis. In addition to the OCR's routine checks, at the end of February 2018, we drew on the Osaka Prefectural electronic resident registration to determine the vital status of patients diagnosed between 2001 and 2002. Patients without death information on this additional check were assumed to be alive. The OCR does not have information on race or ethnicity; however. in 2002, Japanese individuals accounted for 97.7% of the total population of Osaka Prefecture and 90% of the remaining 2.3% were Chinese or Korean.^14^

### 2.2 Inclusion and exclusion criteria

We included female patients diagnosed with first primary breast cancer (ICD‐10, C50) between 2001 and 2002. Additional inclusion criteria were residents of Osaka Prefecture at the time of diagnosis and age at diagnosis between 15 and 85 years. Patients aged 86 years or older were excluded from the analysis because long‐term survival estimates are unstable for older age groups. For patients who had more than one concurrent primary breast cancer, only the cancer with the most comprehensive data was included in our study. In the case of patients who had many subsequent primary breast cancers, we used the data of the earliest diagnosed cancer. In addition, we excluded patients who were only diagnosed by death certificates.

### 2.3 Study cohort

**FIGURE 1:**
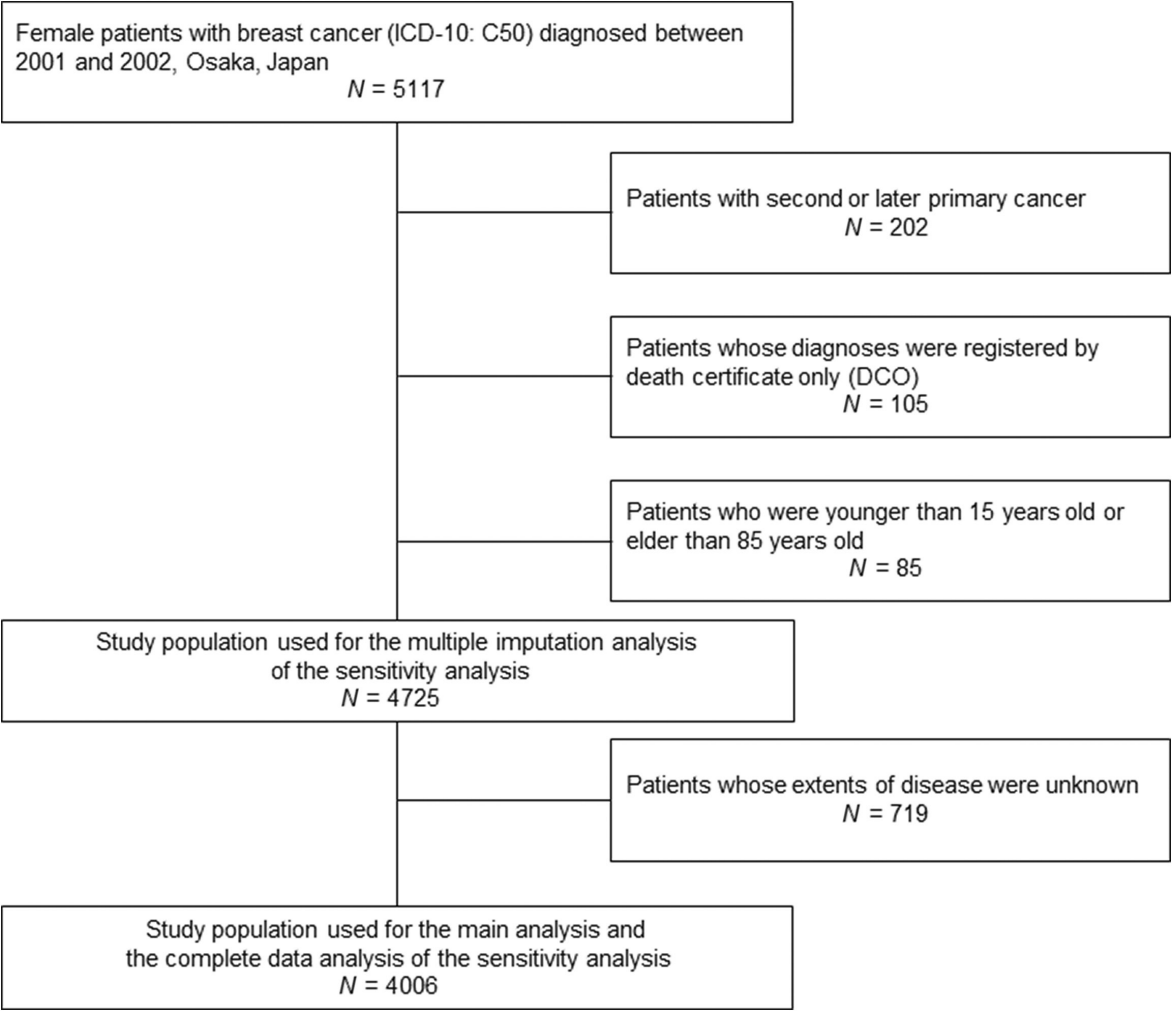
Flow diagram of the study cohort.

Examination of OCR data registered between 2001 and 2002 resulted in identification of 5117 female patients with breast cancer. The following exclusion criteria were applied: second or later primary cancers (*N* = 202); diagnosis registered by death certificate only (*N* = 105); and outside the age criteria (*N* = 85). The remaining 4725 patients were included in the multiple imputation analysis in the sensitivity analysis (described below). In the main and sensitivity analyses of the complete data, we further excluded patients with unknown extent of disease (*N* = 719), leaving 4006 patients (Figure 1).

### 2.4 Statistical method for determining non‐parametric relative survival

First, RS was calculated using a cohort approach after excluding patients whose extent of disease was unknown. RS is the ratio of overall survival of patients to the expected survival of a general population with the same demographic composition. Expected survival is usually obtained from national life tables, which document the survival of the general population, and relies on the assumption that cancer deaths comprise a negligible proportion of all deaths.^15^ The method for calculating the RS is shown in the Data S1. In this study, we used the Ederer II method to calculate RS and national population life tables by single year of age and sex to determine the background mortality of the general population, within the *strs* command in Stata.^16^, ^17^ We also modeled excess mortality using Poisson regression, with the assumption that mortality is constant.^16^ The excess mortality rate ratios (EMRRs; ratios of excess mortality between groups) were determined, and *p* < 0.05 was considered to denote statistical significance. In addition to estimating RS, we calculated age‐standardized relative survival (ASR) by summing the weighted RS according to International Cancer Survival Standard 1^18^ of patients aged <45, 45–54, 55–64, 65–74, and 75–85 years. ASR is a standard internationally recognized variable that is useful for comparing survival between countries with different age distributions.

### 2.5 Sensitivity analysis using a flexible parametric model

As a sensitivity analysis, we calculated RS, ASR, and excess mortality rate with the complete data using a flexible parametric Royston–Parmar model, within the *stpm2* command in Stata.^19^ The excess mortality rate was calculated taking into account time‐dependent effects. In addition, we performed multiple imputation analysis with the study cohort including patients with unknown extent of disease.^20^ We used information on vital status, the Nelson–Aalen estimator of cumulative hazard, and age group for the imputation. Thereafter, 30‐times imputed data were obtained using a multinomial logistic imputation model for chained equations, with the assumption of missing at random mechanism. Using these imputed data, we calculated ASR for all patients with the flexible parametric Royston–Parmar model.

### 2.6 Statistical method for determining conditional relative survival

Using the RS derived by the non‐parametric Ederer‐II method with complete data, we calculated the 5‐year CRS. CRS is defined as the probability of surviving for another α years under the condition of already having lived for *χ* years. Thus, the α‐year CRS for patients who have survived for *χ* years is calculated by dividing the (*χ* + *α*)‐year RS by the χ‐year RS.^12^, ^21^, ^22^, ^23^ We calculated the 5‐year CRS by each year survived up to 10 years after diagnosis. When the 5‐year CRS is 100%, there is no excess mortality among patients with cancer. That is, their survival is comparable to that of the general population. For purposes of analysis, we categorized excess patient mortality relative to the general population as substantial, small, and hardly any when the 5‐year CRS were <90%, 90%–95%, and >95%, respectively.^22^

### 2.7 Variables

We estimated survival for all patients and survival stratified by age group and extent of disease. We created three age groups: young (15–34 years), middle‐aged (35–69), and old (70–85). The cutoff for the youngest age group was defined on the basis of data from studies conducted in Japan and Korea.^24^, ^25^, ^26^, ^27^ We also categorized cancer stage into three groups by extent of disease: localized (Union for International Cancer Control [UICC] classification), T1‐3N0M0; regional (UICC classification, T0‐3N1‐3bM0); and distant metastasis (UICC classification T0‐4N0‐3cM1). We performed the stratified analysis using these variables to determine how survival and excess mortality varied by patient age and extent of disease.

We used Stata version 17 (StataCorp) for all analyses. The dataset was anonymized and analyzed independently in compliance with the Act on Promotion of Cancer Registries. This study was approved by the Research Ethics Committee of the Osaka International Cancer Institute (Approval number: 19143).

## 3 RESULTS

**FIGURE 2:**
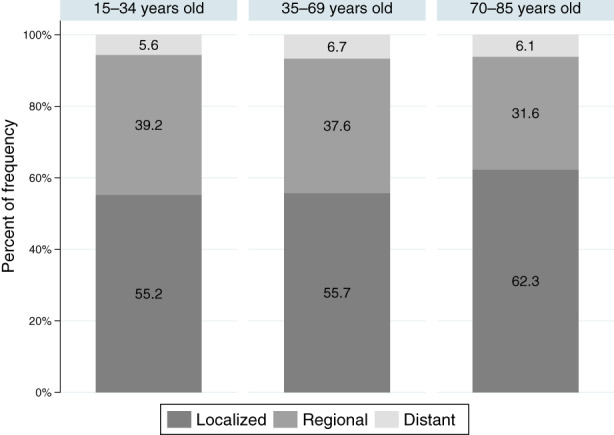
Distribution of extent of disease by age group of 15‐ to 85‐year‐old female patients with first primary breast cancer (ICD‐10: C50) diagnosed between 2001 and 2002, Osaka, Japan (*N* = 4006).

The baseline characteristics of the cohort included in the complete data analysis and the number of patients available for CRS analysis are shown in Table 1. Middle‐aged patients accounted for 80.2% and patients with localized disease for 56.8% of all patients. The distribution of extent of disease varied little between age groups (Figure 2). The baseline characteristics of patients excluded from the complete data analysis were shown in Table S1.

**TABLE 1:** Baseline characteristics of 15‐ to 85‐year‐old female patients with first primary breast cancer (ICD‐10: C50) diagnosed between 2001 and 2002, Osaka, Japan (*N* = 4006).

	*N* (%)	Number of patients available for conditional survival analysis	
		5 years, *N*	10 years, *N*
Total	4006	3332	2860
Age at diagnosis, y			
15–34	125 (3.1)	102	84
35–69	3213 (80.2)	2733	2414
70–85	668 (16.7)	497	362
Extent of disease			
Localized	2276 (56.8)	2104	1914
Regional	1468 (36.7)	1149	914
Distant	262 (6.5)	79	32

### 3.1 Fifteen‐year relative survival and age‐standardized relative survival

**FIGURE 3:**
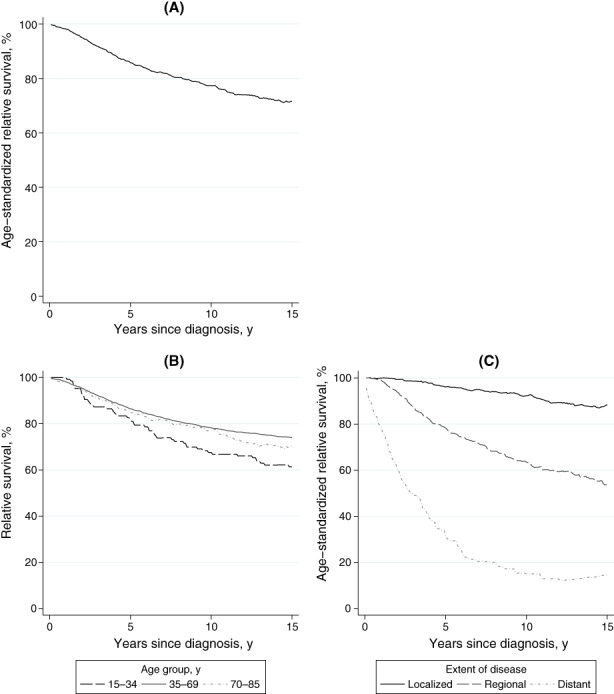
Fifteen‐year relative survival of 15‐ to 85‐year‐old female patients with first primary breast cancer (ICD‐10: C50) diagnosed between 2001 and 2002, Osaka, Japan (*N* = 4006). (A) Age‐standardized relative survival for all patients, (B) Relative survival stratified by age group, (C) Age‐standardized relative survival stratified by extent of disease.

RS, ASR, and adjusted EMRRs are shown in Table 2. Overall, the 15‐year ASR was 71.6%, which is 5.7 percentage points lower than the 10‐year ASR (77.3%; Table 2, Figure 3A). When stratified by age group, the 15‐year RS for young, middle‐aged, and old patients were 61.4%, 74.0%, and 70.3%, respectively (Table 2, Figure 3B). The excess mortality rate adjusted for extent of disease was significantly higher in young than in middle‐aged patients, with an EMRR of 1.62. The 15‐year ASR for patients with localized, regional, and distant disease was 88.4%, 53.5%, and 14.7%, respectively. In patients with regional disease, the 15‐year ASR (53.5%) was 10 percentage points lower than the 10‐year ASR (63.5%) (Table 2, Figure 3C). Excess mortality rates adjusted for age group were significantly higher in patients with regional and distant disease than in patients with localized disease, with EMRRs of 4.47 and 24.39, respectively.

**TABLE 2:** 5‐, 10‐, and 15‐year relative and age‐standardized relative survival by age group and extent of disease for 15‐ to 85‐year‐old female patients with first primary breast cancer (ICD‐10:C50) diagnosed between 2001 and 2002, Osaka, Japan (*N* = 4006).

	Relative survival			EMRR (95%CI)
	5‐year	10‐year	15‐year	
	% (95%CI)	% (95%CI)	% (95%CI)	
All patients^a^	85.8 (83.9–87.7)	77.3 (74.6–80.1)	71.6 (67.9–75.5)	
Age at diagnosis, y				
15–34	81.8 (73.8–87.6)	67.6 (58.5–75.1)	61.4 (52.2–69.4)	1.62* (1.21–2.18)
35–69	86.5 (85.2–87.7)	78.2 (76.6–79.7)	74.0 (72.2–75.7)	Ref.
70–85	85.1 (81.1–88.7)	77.1 (71.6–82.4)	70.3 (62.9–77.6)	1.25 (1.00–1.56)
Extent of disease^a^				
Localized	96.1 (94.1–98.2)	92.3 (89.0–95.7)	88.4 (83.4–93.6)	Ref.
Regional	78.3 (74.7–82.1)	63.5 (59.0–68.4)	53.5 (48.2–59.3)	4.47* (3.69–5.43)
Distant	31.5 (24.4–40.8)	15.0 (9.1–24.6)	14.7 (6.9–31.5)	24.39* (19.63–30.31)

*Note*: The EMRR for each age group was adjusted for extent of disease, and the EMRR for each extent of disease was adjusted for age group.

Abbreviations: CI, confidence interval; EMRR, excess mortality rate ratio.

^a^ The relative survival of all patients and those stratified by extent of disease were age‐standardized, in accordance with the International Cancer Survival Standards.

* Indicates statistically significant (*p < 0.05).

As a sensitivity analysis, we used a flexible parametric model to calculate the ASR and RS with complete data, calculating them with imputed data for patients with unknown extent of disease. The results of the sensitivity analysis are shown in Table S2 and Figure S1–S3. With complete data, the 15‐year ASR for all patients was 72.6% and the 10‐year ASR 77.3%. Excess mortality for each group was highest approximately 4 years after diagnosis, after which declined. Excess mortality persisted to 15 years after diagnosis, especially among young patients and patients with regional and distant disease (Figure S2). We preformed multiple imputation analysis including patients with unknown extent of disease (*N* = 4725, Figure 1). The 15‐year ASR calculated with imputed data was 67.4%, and the 10‐year ASR 72.2% (Table S2, Figure S3). As with the main analysis, the 15‐year survival was lower than the 10‐year survival.

### 3.2 Conditional relative survival

**FIGURE 4:**
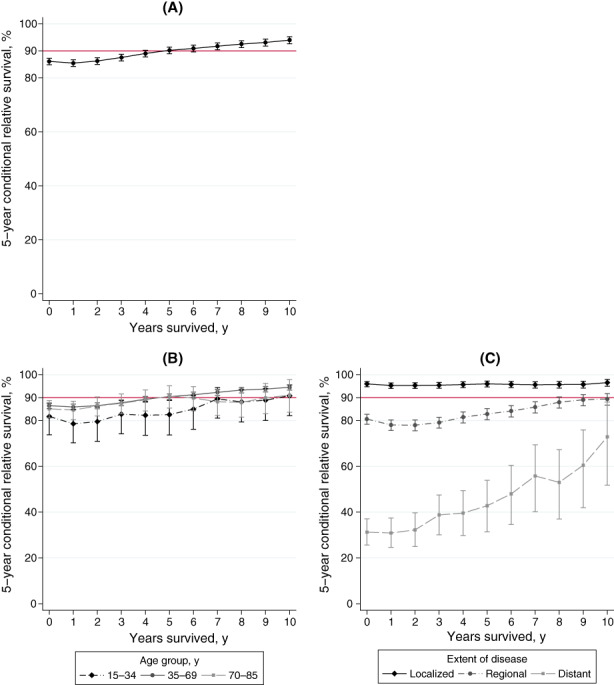
Five‐year conditional relative survival (CRS) of 15‐ to 85‐year‐old female patients with first primary breast cancer (ICD‐10: C50) diagnosed between 2001 and 2002, Osaka, Japan (*N* = 4006). Five‐year CRS are plotted for every additional year survived after the initial diagnosis of cancer. (A) Five‐year CRS for all patients. (B) Five‐year CRS stratified by age group. (C) Five‐year CRS stratified by extent of disease. Red line indicates the 90% border line. Error bars indicate 95% confidence intervals.

In total, the 5‐year CRS was 90.2%, 5 years after diagnosis and 94.0%, 10 years after diagnosis; the 5‐year CRS exceeding 90%, 5 years after diagnosis (Table 3, Figure 4A). The 5‐year CRS 10 years after diagnosis was 90.8% for young patients, 94.5% for middle‐aged patients, and 91.1% for old patients. The 5‐year CRS for middle‐aged or old patients exceeded 90% 5 years after diagnosis; however, it only exceeded 90% for young patients 10 years after diagnosis (Table 3, Figure 4B). In patients with localized disease when diagnosed, the 5‐year CRS was over 95% and 96.5%, 10 years after diagnosis. The 5‐year CRS 10 years after diagnosis in patients with regional and distant disease was 89.4% and 72.9%, respectively, and did not reach 90% during the follow‐up period (Table 3, Figure 4C).

**TABLE 3:** Five‐year conditional relative survival by age group and extent of disease for 15‐ to 85‐year‐old female patients with first primary breast cancer (ICD‐10:C50) diagnosed between 2001 and 2002, Osaka, Japan (*N* = 4006).

	5‐year conditional relative survival at		Conditional relative survival reached 90%
	5‐year after diagnosis	10‐year after diagnosis	
	% (95%CI)	% (95%CI)	At year
All patients	90.2 (88.9–91.4)	94.0 (92.6–95.2)	5
Age at diagnosis, y			
15–34	82.6 (73.7–88.8)	90.8 (82.1–95.5)	10
35–69	90.4 (89.1–91.6)	94.5 (93.3–95.6)	5
70–85	90.6 (85.4–95.2)	91.1 (83.6–97.8)	5
Extent of disease			
Localized	96.0 (94.6–97.2)	96.5 (95.0–97.9)	0
Regional	82.9 (80.3–85.2)	89.4 (86.8–91.8)	—^a^
Distant	42.8 (31.4–53.9)	72.9 (51.8–88.0)	—^a^

Abbreviation: CI, confidence interval.

^a^ Within the available follow‐up period with valid estimates, 5‐year conditional relative survival of >90% was not achieved.

## 4 DISCUSSION

We have here reported 15‐year relative and conditional survival using data drawn from a population‐based cancer registry. The 15‐year ASR for all patients with breast cancer was 71.6%, having declined progressively from the 5‐ and 10‐year ASR. The 5‐year CRS 10 years after diagnosis was 94.0%, indicating that the excess mortality was small (5‐year CRS >90%) from 5 years after diagnosis. Stratified analysis also revealed differences in patterns of relative survival and excess mortality by age group and extent of disease.

Similar to data from other countries, the RS for breast cancer continued to decline beyond 10 years from diagnosis.^28^ The 5‐year CRS for patients with breast cancer continued to increase after diagnosis; however, it did not reach 95% during the 10‐year observation period. In contrast, a previous study reported that the 5‐year CRS for stomach and colorectal cancers exceeded 95% by 5 years post‐diagnosis,^21^ with very little excess mortality. Unlike these cancers, breast cancer still had some excess mortality 10 years after diagnosis. In our study, as in the cited studies, the RS continued to decline beyond 10 years from diagnosis. The 15‐year RS was lower, and the excess mortality rate significantly higher in the young than middle‐aged group, despite the distribution of extent of disease differing only slightly between age groups **(**Tables 2 and 3). Furthermore, in patients with regional or distant disease, the 5‐year CRS did not reach 90% during the 10‐year observation period (89.4% and 72.9%, respectively) and the excess mortality remained significant. One possible explanation for this is late recurrence: in Japan, 70%–80% of all female breast cancer patients have luminal‐like breast cancer,^29^ which is prone to lie dormant for years and lead to late recurrence.^30^ In particular, patients with regional disease^31^, ^32^ and young patients, who are likely to have triple negative cancer,^29^, ^33^ are prone to late recurrences or second cancers.^34^ These findings suggest that early detection and prompt treatment of regional and distant cancer is crucial for improving survival rates. The overall breast cancer screening uptake was 22.5% in 2001 in Japan, compared to 86.9% in the United States in 2000, a fourfold difference.^35^ It was still low 44.6% in 2019, indicating the need for ongoing improvement in the screening rate.^35^

On the basis of these biological and clinical characteristics of breast cancer and the conclusions of the 2001 St. Gallen Conference,^36^ the duration of endocrine therapy was changed from 2 to 5 years in the early 2000s in Japan. More recently, the American Society of Clinical Oncology Clinical Practice Guideline in 2019 recommended continuing adjuvant endocrine therapy for 10 years.^11^ In this study, we found that young patients and patients with regional and distant disease diagnosed during 2001–2002 in Japan may need to be followed up for longer than 10 years, even though the effect of these recent changes in treatment strategy is unknown. The present findings suggest that surveillance methods and duration of endocrine therapy need to be adjusted according to the patient's age group and stage of disease.

We compared the 15‐year survival and CRS obtained in this study with those reported from Western countries. The reported 15‐year ASR in the Netherlands is comparable to that in our study (66.0% for ages 18 years and over, 2000–2009).^28^ In contrast, the 15‐year RS was 80.3% in 2001 in the United States.^37^ Although it is problematic to simply compare ASR and not‐age‐standardized RS, differences in the general population's life expectancy is one possible reason for the differences in RS, which were calculated using life tables. In 2001, Japanese women had a longer life expectancy (84.9 years) than did women in the United States (79.8 years).^38^, ^39^ In Japan, the 5‐year overall CRS 10 years after diagnosis (93.5%) is similar to that reported for Australia (93.3% in 1998–2006 for 15‐ to 89‐year‐old patients).^40^ Additionally, that for young patients (91.3%) was comparable to that reported for Europe (91% in 1985–2004 for 15‐ to 44‐year‐old patients)^22^ and the United States (93.3% in 1973–2009 for 15‐ to 39‐year‐old patients).^41^ The excess mortality for each age group is also comparable to that reported in Europe,^22^ where excess mortality for young patients declines later than that in middle‐aged patients.

### 4.1 Strengths

One of the strengths of our study is that we calculated the 15‐year survival by a cohort approach, using Japanese population‐based cancer registry data with long‐term follow‐up. A second strength is that we used high‐quality OCR data: the OCR has complied with the International Agency for Research on Cancer's standards for comparability and quality for more than 40 years.^42^

### 4.2 Limitations

Several limitations of our study are of concern. First, the obtained data lacked information on socioeconomic status, race, ethnicity, and clinical characteristics, such as tumor subtype (including hormone receptor status), recurrence, cause of death, comorbidities, and drugs administered. Some cancer registries, such as the Surveillance, Epidemiology, and End Results in the USA, include clinical data that the OCR does not. Now, in the era of treatment based on receptor status, our cancer registry may need to routinely collect or link such crucial clinical data. Second, the data were collected from a single prefecture's cancer registry; however, Osaka is the third largest prefecture in Japan. Because of this, only a small number of cases were available for stratified analysis, which may limit the applicability of the results to all Japanese women who have had breast cancer. Third, changes in standard therapy occurred during the follow‐up period. The duration of treatment in this study may also have varied because of changes in management guidelines. Furthermore, the results of this study do not indicate a causal relationship with the duration of treatment. Some of these limitations will have been addressed with the launch of the national cancer registry in 2016 and the ongoing follow‐up of patients. Currently, however, data with long‐term follow‐up of more than 10 years in Japan are limited, making the findings of this study valuable.

## 5 CONCLUSIONS

In our study cohort, survival of patients with breast cancer continued to decline beyond 10 years from diagnosis, there being some degree of excess mortality even 10 years after diagnosis, especially in young patients and patients with regional and distant disease. For these patients, physician follow‐up may be necessary for 10 or more years after diagnosis. In addition, the need for long‐term survivorship support from the government and society is suggested. For these reasons, data from long‐term follow‐up would be valuable to patients in planning their lives, to clinicians in planning their treatment, and to the government in considering policies for supporting patients.

## AUTHOR CONTRIBUTIONS

**Mizuki Kato:** Conceptualization (equal); data curation (lead); formal analysis (lead); investigation (lead); methodology (lead); project administration (lead); software (lead); visualization (lead); writing – original draft (lead). **Kayo Nakata:** Conceptualization (supporting); formal analysis (supporting); investigation (supporting); methodology (supporting); project administration (supporting); visualization (supporting); writing – original draft (supporting). **Toshitaka Morishima:** Validation (supporting); writing – original draft (supporting); writing – review and editing (supporting). **Yoshihiro Kuwabara:** Writing – review and editing (supporting). **Fumie Fujisawa:** Writing – review and editing (supporting). **Nobuyoshi Kittaka:** Writing – review and editing (supporting). **Takahiro Nakayama:** Writing – review and editing (supporting). **Isao Miyashiro:** Supervision (lead); writing – review and editing (supporting).

## FUNDING INFORMATION

This work was supported by a grant from the Osaka Foundation for Prevention of Cancer and Circulatory Diseases in 2021 and by Health Labour and Welfare Sciences Research Grants (H30‐Gantaisaku Ippan‐009) from the Ministry of Health, Labour and Welfare, Japan.

## CONFLICT OF INTEREST STATEMENT

All authors have no conflicts of interest.

## ETHICAL APPROVAL STATEMENT

This study was approved by the Research Ethics Committee of the Osaka International Cancer Institute (Approval number: 19143).

## Supporting information


Data S1

Figure S1–S3

Table S1–S2

## Data Availability

The data that support the findings of this study are available on request from the corresponding author. The data are not publicly available due to privacy or ethical restrictions.
