# Supplementary material for: Fifteen‐year survival and conditional survival of women with breast cancer in Osaka, Japan: A population‐based study
Source: Cancer Med. 2023 May 4;12(12):13774–83. doi: 10.1002/cam4.6016 (PMC10315741; doi:10.1002/cam4.6016)
Supplement: Supplementary file 1 — Data S1 [file CAM4-12-13774-s003.docx]

For patients diagnosed with cancer, the total hazard rate, $h\left( t \right)$ is considered as the sum of the expected mortality rate due to other causes, $h^{*}\left( t \right)$, and the excess mortality rate associated with cancer, $\lambda(t)$:

$$h\left( t \right)=h^{*}\left( t \right)+\lambda\left( t \right) (1)$$

Based on the assumption that the cancer deaths are a negligible proportion of all deaths, the expected mortality rate is usually obtained from life tables, which represents the survival of the general population. (Cho. et al., 2011) The functions of definitions about the cumulative hazard function, $H(t)$, and the survival function, $S(t)$, are as follows:

$$H\left( t \right)=\int_{0}^{t} h\left( u \right)dt$$

$$S\left( t \right)=exp\{-H\left( t \right)\}$$

If we transform the equation (1) using these definitions, the overall survival, $S(t)$ , can be obtained by the multiplication of the expected survival, $S^{*}(t)$, and the survival associated with cancer, $R(t)$ :

$$S\left( t \right)=S^{*}\left( t \right)R(t)$$

$R(t)$ , referred to as relative survival, can be obtained as the ratio of the overall survival to expected survival:

$$R(t)=\frac{S(t)}{S^{*}(t)}$$

In this study, we derived RS using national population life tables by single year of age and sex to determine the background mortality of the general population.

**Reference**

CHO., H., HOWLADER., N., MARIOTTO., A. B. & CRONIN., K. A. 2011. Estimating relative survival for cancer patients from the SEER Program using expected rates based on Ederer I versus Ederer II method. *Surveillance Research Program, NCI, Technical Report,* 01.
