# Supplementary material for: Fifteen‐year survival and conditional survival of women with breast cancer in Osaka, Japan: A population‐based study
Source: Cancer Med. 2023 May 4;12(12):13774–83. doi: 10.1002/cam4.6016 (PMC10315741; doi:10.1002/cam4.6016)
Supplement: Supplementary file 2 — Figure S1–S3 [file CAM4-12-13774-s001.docx]

**
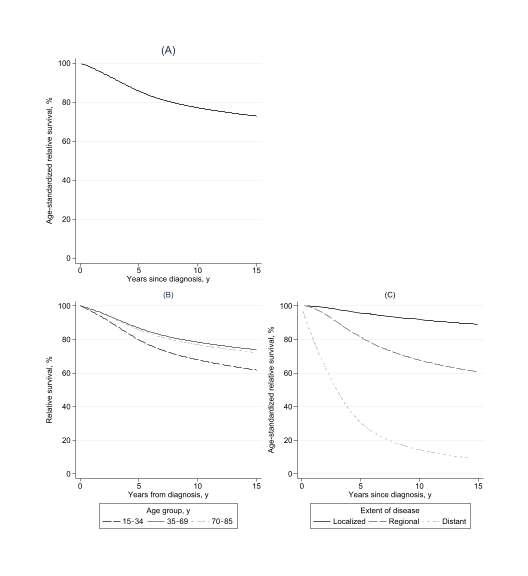
**

**Supplementary Figure 1.**

**Fifteen-year relative survival of 15–85-year-old female patients with first primary breast cancer (ICD-10: C50) diagnosed between 2001 and 2002, Osaka, Japan (N=4,006). Calculated using flexible parametric Royston Parmar model.**

(A) Age-standardized relative survival for all patients. (B) Relative survival stratified by age group. (C) Age-standardized relative survival stratified by extent of disease.

**
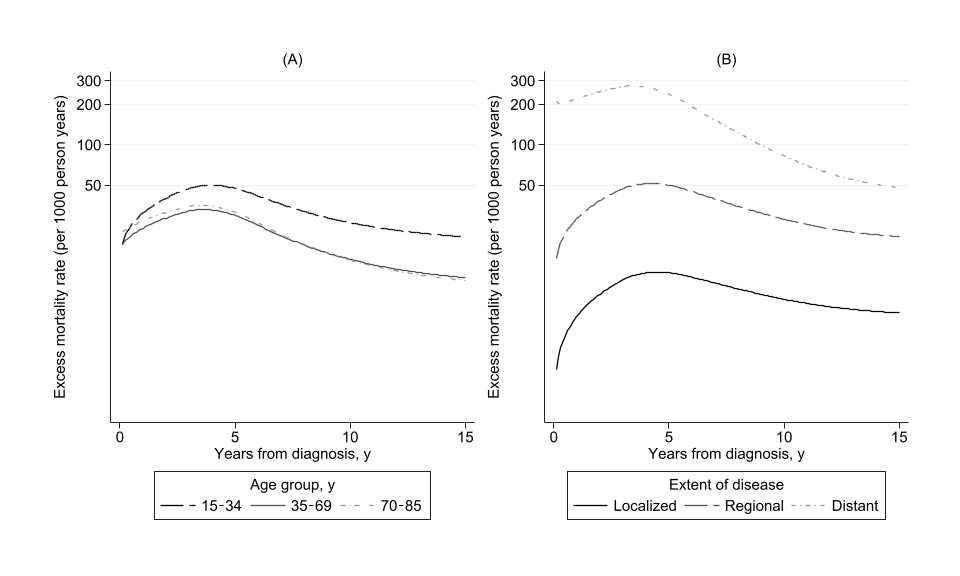
**

**Supplementary Figure 2.**

**Excess mortality rate of 15–85-year-old female patients with first primary breast cancer (ICD-10: C50) diagnosed between 2001 and 2002, Osaka, Japan (N=4,006).**

1. Excess mortality rate for each age group. (B) Excess mortality rate for each extent of disease.

**
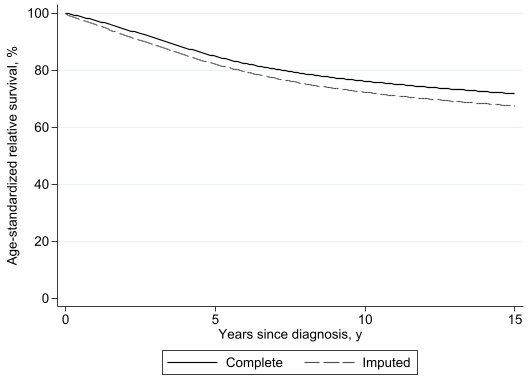
Supplementary Figure 3.**

**Fifteen-year age-standardized relative survival of 15–85-year-old female patients with first primary breast cancer (ICD-10: C50) diagnosed between 2001 and 2002, Osaka, Japan (N=4,725). Multiple imputation was performed for patients with missing extent of disease (N=725).**

The estimation obtained by the complete data analysis, which was calculated without the patients with missing data, is shown as “Complete.” The estimation obtained by multiple imputation for patients with missing data is shown as “Imputed”.
