# Supplementary material for: Fifteen‐year survival and conditional survival of women with breast cancer in Osaka, Japan: A population‐based study
Source: Cancer Med. 2023 May 4;12(12):13774–83. doi: 10.1002/cam4.6016 (PMC10315741; doi:10.1002/cam4.6016)
Supplement: Supplementary file 3 — Table S1–S2 [file CAM4-12-13774-s002.docx]

**Supplementary Table 1.**

**Baseline characteristics of excluded female patients with breast cancer (ICD-10: C50) diagnosed between 2001 and 2002, Osaka, Japan**

|  |  |  |  | Number of patients who survived for | | |
| --- | --- | --- | --- | --- | --- | --- |
|  |  | N (%) |  | 5 years, N |  | 10 years, N |
| **Patients with second or later primary cancer** | | | | |  |  |
| Total |  | 194 |  | 133 |  | 105 |
| Age at diagnosis, y |  |  |  |  |  |  |
| 15–34 | | 0 |  | 0 |  | 0 |
| 35–69 | | 127 (65.5) |  | 94 |  | 79 |
| 70–85 | | 67 (34.5) |  | 39 |  | 26 |
| Extent of disease |  |  |  |  |  |  |
| Localized |  | 108 (55.7) |  | 86 |  | 70 |
| Regional |  | 40 (20.6) |  | 27 |  | 19 |
| Distant |  | 11 (5.7) |  | 4 |  | 2 |
| Unknown |  | 35 (18.0) |  | 16 |  | 14 |
| **Patients whose diagnoses were registered by death certificate only (DCO)** | | | | |  |  |
| Total |  | 92 |  | 0 |  | 0 |
| Age at diagnosis, y |  |  |  |  |  |  |
| 15–34 | | 0 |  | 0 |  | 0 |
| 35–69 | | 53 (57.6) |  | 0 |  | 0 |
| 70–85 | | 39 (42.4) |  | 0 |  | 0 |
| Extent of disease |  |  |  |  |  |  |
| Localized |  | 0 |  | 0 |  | 0 |
| Regional |  | 0 |  | 0 |  | 0 |
| Distant |  | 0 |  | 0 |  | 0 |
| Unknown |  | 92 (100.0) |  | 0 |  | 0 |
| **Patients who were younger than 15 years old or elder than 85 years old** | | | | |  |  |
| Total |  | 113 |  | 34 |  | 17 |
| Extent of disease |  |  |  |  |  |  |
| Localized |  | 38 (33.6) |  | 25 |  | 14 |
| Regional |  | 13 (11.5) |  | 5 |  | 1 |
| Distant |  | 7 (6.2) |  | 1 |  | 1 |
| Unknown |  | 55 (48.7) |  | 3 |  | 1 |
| **Patients whose extents of disease were unknown** | | | | | |  |
| Total |  | 839 |  | 494 |  | 363 |
| Age at diagnosis, y |  |  |  |  |  |  |
| 15–34 | | 27 (3.2) |  | 21 |  | 15 |
| 35–69 | | 642 (76.5) |  | 418 |  | 314 |
| 70–85 | | 170 (20.3) |  | 55 |  | 34 |

**Supplementary Table 2.**

**Five-, 10-, and 15-year relative and age-standardized relative survival by age group and extent of disease for 15-85-year-old female patients with primary first breast cancer (ICD-10:C50) diagnosed between 2001 and 2002, Osaka, Japan(N=4,006). Analyzed using flexible parametric Royston-Parmar model**

|  |  | Relative Survival | | | | |  |  |
| --- | --- | --- | --- | --- | --- | --- | --- | --- |
|  |  | 5-year |  | 10-year |  | 15-year |  |  |
|  |  | % (95%CI) |  | % (95%CI) |  | % (95%CI) |  | EMRR (95%CI) |
| All patients ^a^ |  | 86.1 (84.5–87.8) |  | 77.3 (74.9–79.8) |  | 72.6 (69.8–75.6) |  |  |
| All patients ^a^  (imputed) ^b^ | | 82.1 (80.7–83.5) |  | 72.2 (70.4–74.0) |  | 67.4 (65.3–69.5) |  |  |
| Age at diagnosis, y |  |  |  |  |  |  |  |  |
| 15–34 |  | 79.8 (74.0–84.5) |  | 67.9 (59.7–74.8) |  | 61.8 (52.7–69.6) |  | 1.62* (1.20–2.17) |
| 35–69 |  | 86.8 (85.6–87.9) |  | 78.4 (76.8–79.9) |  | 73.9 (72.2–75.6) |  | Reference |
| 70–85 |  | 85.8 (82.4–88.6) |  | 76.9 (71.8–81.2) |  | 72.2 (66.2–77.3) |  | 1.21 (0.96–1.51) |
| Extent of disease ^a^ | | |  |  |  |  |  |  |
| Localized |  | 95.9 (95.0–96.9) |  | 91.8 (90.5–93.2) |  | 89.0 (87.3–90.8) |  | Reference |
| Regional |  | 81.4 (79.4–83.5) |  | 67.7 (65.2–70.3) |  | 60.7 (57.9–63.6) |  | 4.44* (3.68–5.35) |
| Distant |  | 30.6 (25.7–36.4) |  | 14.3 (10.8–18.9) |  | 8.8 (5.8–13.4) |  | 24.46* (19.78–30.24) |

Abbreviations: CI, confidence interval; EMRR, excess mortality rate ratio
^a^: Relative survival of all patients and those stratified by extent of disease were age-standardized, according to the International Cancer Survival Standards.

^b^: Survival was calculated with multiple imputation for the patients with unknown extent of disease, who were excluded from the main analysis. There were 4,725 patients in the cohort for this calculation.

* indicates statistical significance (*P < 0.05).
